# Analysis of survival after initiation of continuous renal replacement therapy in patients with extracorporeal membrane oxygenation

**DOI:** 10.1186/s12882-019-1516-6

**Published:** 2019-08-14

**Authors:** George Kuo, Shao-Wei Chen, Pei-Chun Fan, Victor Chien-Chia Wu, An-Hsun Chou, Cheng-Chia Lee, Pao-Hsien Chu, Feng-Chun Tsai, Ya-Chung Tian, Chih-Hsiang Chang

**Affiliations:** 1 Department of Nephrology, Kidney Research Center, Chang Gung Memorial Hospital, Linkou Medical Center, College of Medicine, Chang Gung University, No.5, Fuxing Street, Guishan District, Taoyuan City, Taiwan 33305; 2 Department of Cardiothoracic and Vascular Surgery, Chang Gung Memorial Hospital, Linkou Medical Center, Chang Gung University, Taoyuan, Taiwan; 3 Department of Cardiology, Chang Gung Memorial Hospital, Linkou Medical Center, Chang Gung University, Taoyuan, Taiwan; 4 Department of Anesthesiology, Chang Gung Memorial Hospital, Linkou Medical Center, Chang Gung University, Taoyuan, Taiwan

**Keywords:** Continuous renal replacement therapy, Extracorporeal membrane oxygenation, Acute kidney injury, Mortality

## Abstract

**Background:**

No study has specifically investigated the duration of continuous renal replacement therapy (CRRT) in patients who experienced acute kidney injury during extracorporeal membrane oxygenation (ECMO) support. However, there are concerns that prolonged CRRT may be futile.

**Methods:**

We conducted a retrospective population-based cohort study using Taiwan National Health Insurance Research Database data collected between January 1, 2007 and December 31, 2013. Patients who received ECMO and CRRT during the study period were included. We divided patients into three groups based on the duration of CRRT received: ≤ 3 days, 4–6 days, and ≥ 7 days. The outcomes were all-cause mortality, end-stage renal disease, ventilator dependency, and readmission rate.

**Results:**

There were 247, 134 and 187 patients who survived the hospitalization in the CRRT for ≤3 days, 4–6 days and > 7 days respectively. Survival after discharge did not differ significantly between CRRT for 4–6 days vs. ≤ 3 days (adjusted hazard ratio [aHR] 1.16, 95% confidence interval [CI] 0.85–1.57), between CRRT for > 7 days vs. ≤ 3 days (aHR 1.001, 95% CI 0.73–1.38) and between CRRT for > 7 days vs. 4–6 days (aHR 0.87, 95% CI 0.62–1.22). The patients who received CRRT for ≥7 days had a higher risk of ESRD than did those who received CRRT for ≤3 days (adjusted hazard ratio [aHR] 3.46, 95% confidence interval [CI] 1.47–8.14) and for 4–6 days (aHR 3.10, 95% CI 1.03–9.29). The incidence of ventilator dependence was higher in the patients with CRRT ≥7 days than in those with ≤3 days (aHR 2.45, 95% CI 1.32–4.54). The CRRT ≥7 days group also exhibited a higher readmission rate than did the 4–6 days and ≤ 3 days groups (aHR 1.43, 95% CI 1.04–1.96 and aHR 1.67, 95% CI 1.13–2.47, respectively).

**Conclusions:**

Our study found similar long-term survival but increased ESRD and ventilator dependency among ECMO patients who underwent CRRT for ≥7 days. These results offer reason to be concerned that this aggressive life support may maintain patient survival but do so at the cost of long-term disabilities and a lower quality of life.

**Electronic supplementary material:**

The online version of this article (10.1186/s12882-019-1516-6) contains supplementary material, which is available to authorized users.

## Background

Extracorporeal membrane oxygenation (ECMO) has been utilized for both circulatory and respiratory support in critical care for the past two decades, including for patients with cardiogenic shock, post-cardiotomy shock, acute respiratory distress syndrome, and trauma [1–6]. Acute kidney injury (AKI) is frequently observed in patients who require ECMO support, and the presence of AKI is associated with higher mortality [7–9]. Continuous renal replacement therapy (CRRT) benefits patients with renal failure who are too hemodynamically unstable for intermittent hemodialysis. Thus, CRRT is frequently utilized to treat AKI in patients receiving ECMO support.

Concerns have been raised regarding the optimal duration of CRRT in critically ill patients; prolonged use of life support therapy may be futile if the underlying condition is not corrected [10]. However, because of the lack of long-term follow-up data and the fact that few patients survive within each single center, little is known about how CRRT duration affects the outcomes of patients receiving ECMO. More evidence is required to accurately assess the long-term outcomes in order to potentially justify the cost-effectiveness of prolonged life support therapies such as ECMO and CRRT.

No previous study has reported the long-term outcomes of patients who received ECMO and CRRT. Taiwan’s National Health Insurance Research Database (NHIRD) provides a means of investigating this issue because of the high coverage rate of the National Health Insurance program and its reimbursement data for high-cost life-support therapies, including ECMO and CRRT. Therefore, we used NHIRD data to conduct a population-based cohort study aimed at elucidating the long-term mortality, morbidity, readmission, and expenditure of patients who received ECMO with different durations of CRRT in Taiwan.

## Methods

### Data source and study population

We performed this study by analyzing data from Taiwan’s NHIRD, a nationwide research database containing no identifiable personal information (see description of NHIRD in Additional file 1: materials). The study was approved by the Institutional Review Board of Chang Gung Memorial Hospital, and the need for informed consent was waived because of this study’s retrospective, non-interventional design and because patient data confidentiality and privacy were maintained.

We retrieved data from patients who were recorded in the inpatient claims database as receiving first-time ECMO between January 1, 2007 and December 31, 2013. The admission date was defined as the index date. We excluded patients who were younger than 20 years, who had end-stage renal disease (ESRD) before the index date, who survived less than 24 h after the initiation of ECMO, or who did not undergo CRRT. To evaluate the effect of dialysis duration on the long-term outcomes of patients receiving ECMO, we divided the patients into three groups based on the duration of CRRT received: 3 days or less, 4–6 days, and 7 days or more, per Tatum et al. [10].

### Definitions of variables

The patients’ demographic information was recorded at the index date. Their comorbidities were defined using the International Classification of Diseases, Ninth Revision, Clinical Modification (ICD-9-CM) diagnostic codes of medical records before index admission (see Additional file 1: Table S1). The use of ECMO was identified by the presence of an ICD-9-CM procedure code (39.65) and the reimbursement code used for ECMO in the NHIRD [2]. Indications of ECMO were determined using the NHI reimbursement code for the procedure and an appropriate ICD-9-CM diagnostic code (e.g., myocardial infarction, cardiogenic shock, respiratory failure, pneumonia, or trauma) [11]. The use of CRRT was identified with the reimbursement code of continuous veno-venous hemodialysis (CVVHD), continuous veno-venous hemofiltration (CVVH), or continuous arterio-venous hemofiltration (CAVH). The duration of CRRT was determined by the amount of reimbursement for CVVHD, CVVH, or CAVH.

The primary outcomes of this study were all-cause mortality, ESRD, ventilator dependency, and readmission for any cause. Mortality was identified by withdrawal from the NHI [12]. Both ESRD and ventilator dependency were identified by the presence of an application for a catastrophic illness certificate after the index date (see the Additional file 1: materials for a description of the certificates). All patients were followed from the index date to the date of event occurrence, date of death, or December 31, 2013.

### Statistical analysis

Continuous data were expressed as mean ± standard deviation (SD) and categorical data were expressed as frequency and percentage (%). Differences among the study groups (groups by CRRT duration) were compared using the chi-square test and one-way analysis of variance for categorical and continuous variables, respectively. Bonferroni multiple comparisons were used for pairwise comparisons between any two CRRT groups. We further analyzed long-term outcomes, namely all-cause mortality, ESRD, ventilator dependency, and readmission, in the patients who survived the index hospitalization. We performed Cox proportional hazard model analysis on all-cause mortality and used subdistribution hazard models (SDHs) to examine the other time-to-event outcomes, with death being considered a competing risk. Both the Cox and SDH models were adjusted for the baseline characteristics listed in Table 1 except for follow-up years (models 2–4 in Table 2). The results obtained from model 3 of Table 2 were considered the primary analysis. We additionally adjusted for some in-hospital outcomes (i.e., the presence of sepsis, respiratory failure, and massive blood transfusion during hospitalization; the length of hospital and ICU stay; and duration on ECMO and ventilator) in model 4 of Table 2. A two-sided *P* value of < 0.05 was considered statistically significant. Data analysis was conducted using SAS software version 9.4 (SAS Institute, Cary, NC).

**Table 1:** Baseline patient profiles (*n* = 2272)

Variable	CRRT ≤3 days (*n* = 1234)	CRRT 4–6 days (*n* = 451)	CRRT ≥7 days (*n* = 587)	*P*
Age (years)	57.7 ± 15.8	58.3 ± 16.0	57.5 ± 15.8	0.696
Age group (years)				0.443
≤ 40	190 (15.4)	62 (13.7)	94 (16.0)	
41–50	179 (14.5)	70 (15.5)	91 (15.5)	
51–60	285 (23.1)	102 (22.6)	123 (21.0)	
61–70	252 (20.4)	101 (22.4)	140 (23.9)	
71–80	256 (20.7)	85 (18.8)	96 (16.4)	
> 80	72 (5.8)	31 (6.9)	43 (7.3)	
Sex				0.689
Male	895 (72.5)	319 (70.7)	417 (71.0)	
Female	339 (27.5)	132 (29.3)	170 (29.0)	
ECMO indication				0.007
CV (cardiogenic shock, myocarditis, or AMI)	341 (27.6)	107 (23.7)	117 (19.9)^a^	
Post-cardiotomy shock	545 (44.2)	219 (48.6)	285 (48.6)	
Respiratory	256 (20.7)	94 (20.8)	154 (26.2)^a^	
Trauma	54 (4.4)	20 (4.4)	19 (3.2)	
Other	38 (3.1)	11 (2.4)	12 (2.0)	
ECMO duration (days)	3.9 ± 4.1	5.2 ± 3.9^a^	7.1 ± 6.4^a^	< 0.001
Comorbidities				
Chronic kidney disease	152 (12.3)	62 (13.7)	81 (13.8)	0.588
Diabetes mellitus	367 (29.7)	141 (31.3)	203 (34.6)	0.114
Hypertension	496 (40.2)	180 (39.9)	228 (38.8)	0.858
Heart failure	267 (21.6)	86 (19.1)	102 (17.4)	0.089
Coronary artery disease	650 (52.7)	238 (52.8)	282 (48.0)	0.151
Prior myocardial infarction	178 (14.4)	46 (10.2)	67 (11.4)	0.036
Atrial fibrillation	125 (10.1)	37 (8.2)	63 (10.7)	0.371
Peripheral arterial disease	50 (4.1)	25 (5.5)	26 (4.4)	0.421
Prior stroke	124 (10.0)	51 (11.3)	67 (11.4)	0.596
Chronic obstructive pulmonary disease	81 (6.6)	28 (6.2)	41 (7.0)	0.880
Liver cirrhosis	62 (5.0)	18 (4.0)	27 (4.6)	0.668
Malignancy	79 (6.4)	25 (5.5)	40 (6.8)	0.700
Charlson score	2.63 ± 2.28	2.52 ± 2.17	2.65 ± 2.30	0.619
Study year				< 0.001
2007–2009	412 (33.4)	124 (27.5)	149 (25.4)^a^	
2010–2011	409 (33.1)	141 (31.3)	182 (31.0)	
2012–2013	413 (33.5)	186 (41.2)^a^	256 (43.6)^a^	
ECMO volume				0.071
1st (1–143)	327 (26.5)	125 (27.7)	149 (25.4)	
2nd (149–220)	268 (21.7)	120 (26.6)	144 (24.5)	
3rd (230–441)	264 (21.4)	102 (22.6)	118 (20.1)	
4th (485–719)	375 (30.4)	104 (23.1)	176 (30.0)	
Hospital level				< 0.001
Medical center	879 (71.2)	346 (76.7)	483 (82.3)^a^	
District/regional hospital	355 (28.8)	105 (23.3)	104 (17.7)^a^	
Follow-up (years)	0.36 ± 1.09	0.50 ± 1.33	0.44 ± 1.08	0.062

*AMI* Acute myocardial infarction, *CRRT* Continuous renal replacement therapy, *CV* Cardiovascular, *ECMO* Extracorporeal membrane oxygenation

^a^indicates *P* < 0.05 versus CRRT ≤3 days in the Bonferroni multiple comparisons

**Table 2:** Follow-up outcomes of patients who survived the index hospitalization (*n* = 568)

	Event (%)			Adjusted HR (95% CI), *P* value					
	≤ 3 days	4–6 days	≥ 7 days	4–6 days vs. ≤ 3 days		≥ 7 days vs. ≤ 3 days		≥ 7 days vs. 4–6 days	
Outcome/model	(*n* = 247)	(*n* = 134)	(*n* = 187)	aHR (95% CI)	*P*	aHR (95% CI)	*P*	aHR (95% CI)	*P*
All-cause mortality									
Model 1	139 (56.3)	78 (58.2)	90 (48.1)	1.12 (0.84–1.49)	0.456	0.97 (0.74–1.28)	0.832	0.87 (0.63–1.20)	0.390
Model 2	139 (56.3)	78 (58.2)	90 (48.1)	1.19 (0.89–1.60)	0.245	0.94 (0.71–1.25)	0.684	0.79 (0.57–1.10)	0.162
Model 3	139 (56.3)	78 (58.2)	90 (48.1)	1.15 (0.86–1.55)	0.348	0.91 (0.68–1.22)	0.536	0.79 (0.57–1.10)	0.164
Model 4	139 (56.3)	78 (58.2)	90 (48.1)	1.16 (0.85–1.57)	0.353	1.001 (0.73–1.38)	0.993	0.87 (0.62–1.22)	0.409
Long-term dialysis^a^									
Model 1	16 (6.5)	8 (6.0)	17 (9.1)	0.90 (0.34–2.41)	0.831	2.64 (1.29–5.40)	0.008	2.94 (1.14–7.57)	0.025
Model 2	16 (6.5)	8 (6.0)	17 (9.1)	0.93 (0.33–2.64)	0.890	2.80 (1.33–5.90)	0.007	3.01 (1.11–8.17)	0.030
Model 3	16 (6.5)	8 (6.0)	17 (9.1)	1.12 (0.40–3.14)	0.834	3.46 (1.47–8.14)	0.005	3.10 (1.03–9.29)	0.044
Model 4	16 (6.5)	8 (6.0)	17 (9.1)	0.93 (0.34–2.58)	0.891	2.30 (0.78–6.75)	0.131	2.47 (0.76–7.97)	0.131
Ventilator dependent^a^									
Model 1	23 (9.3)	17 (12.7)	34 (18.2)	1.32 (0.69–2.55)	0.404	2.47 (1.39–4.40)	0.002	1.87 (0.99–3.52)	0.052
Model 2	23 (9.3)	17 (12.7)	34 (18.2)	1.37 (0.70–2.70)	0.357	2.50 (1.39–4.47)	0.002	1.82 (0.97–3.42)	0.064
Model 3	23 (9.3)	17 (12.7)	34 (18.2)	1.28 (0.64–2.54)	0.485	2.45 (1.32–4.54)	0.004	1.92 (0.99–3.72)	0.054
Model 4	23 (9.3)	17 (12.7)	34 (18.2)	1.23 (0.60–2.52)	0.580	1.73 (0.87–3.43)	0.116	1.41 (0.70–2.84)	0.333
Readmission^a^									
Model 1	92 (37.2)	47 (35.1)	83 (44.4)	0.88 (0.62–1.26)	0.494	1.43 (1.05–1.94)	0.024	1.62 (1.11–2.36)	0.013
Model 2	92 (37.2)	47 (35.1)	83 (44.4)	0.86 (0.60–1.26)	0.443	1.43 (1.05–1.94)	0.023	1.65 (1.12–2.44)	0.012
Model 3	92 (37.2)	47 (35.1)	83 (44.4)	0.85 (0.59–1.24)	0.411	1.43 (1.04–1.96)	0.028	1.67 (1.13–2.47)	0.010
Model 4	92 (37.2)	47 (35.1)	83 (44.4)	0.82 (0.56–1.20)	0.307	1.08 (0.77–1.51)	0.668	1.31 (0.87–1.98)	0.197

*aHR* adjusted hazard ratio, *CI* Confidence interval

Model 1 was adjusted for baseline age, sex, comorbidities listed in Table 1, and study year; Model 2 was further adjusted for baseline monthly income and urbanization level of area of residence; Model 3 was further adjusted for ECMO indication, ECMO volume, and hospital level; Model 4 was further adjusted for in-hospital outcomes, including sepsis, respiratory failure, massive blood transfusion, ECMO duration (days), ventilator days, ICU duration, and hospital stay length;

^a^was estimated using a subdistribution hazard model, with death being considered as a competing risk

### Validation

To verify the accuracy of our primary variables, a chart review cross-comparison was performed. The validation was conducted using a chart review of patients who received first-time ECMO treatment between January 2011 and December 2012 in a tertiary medical center in Taiwan, namely Chang Gung Memorial Hospital, Taoyuan. The patients in the chart review were linked to those in the NHIRD based on date of birth, sex, admission date, and discharge date. We validated the indications of ECMO and the use of CRRT. After linking the two sources, we obtained the positive predictive values and negative predictive values of ECMO indications and the use of CRRT between the chart review and the NHIRD (see Additional file 1: Figure S2 and S3).

## Results

**Fig. 1:**
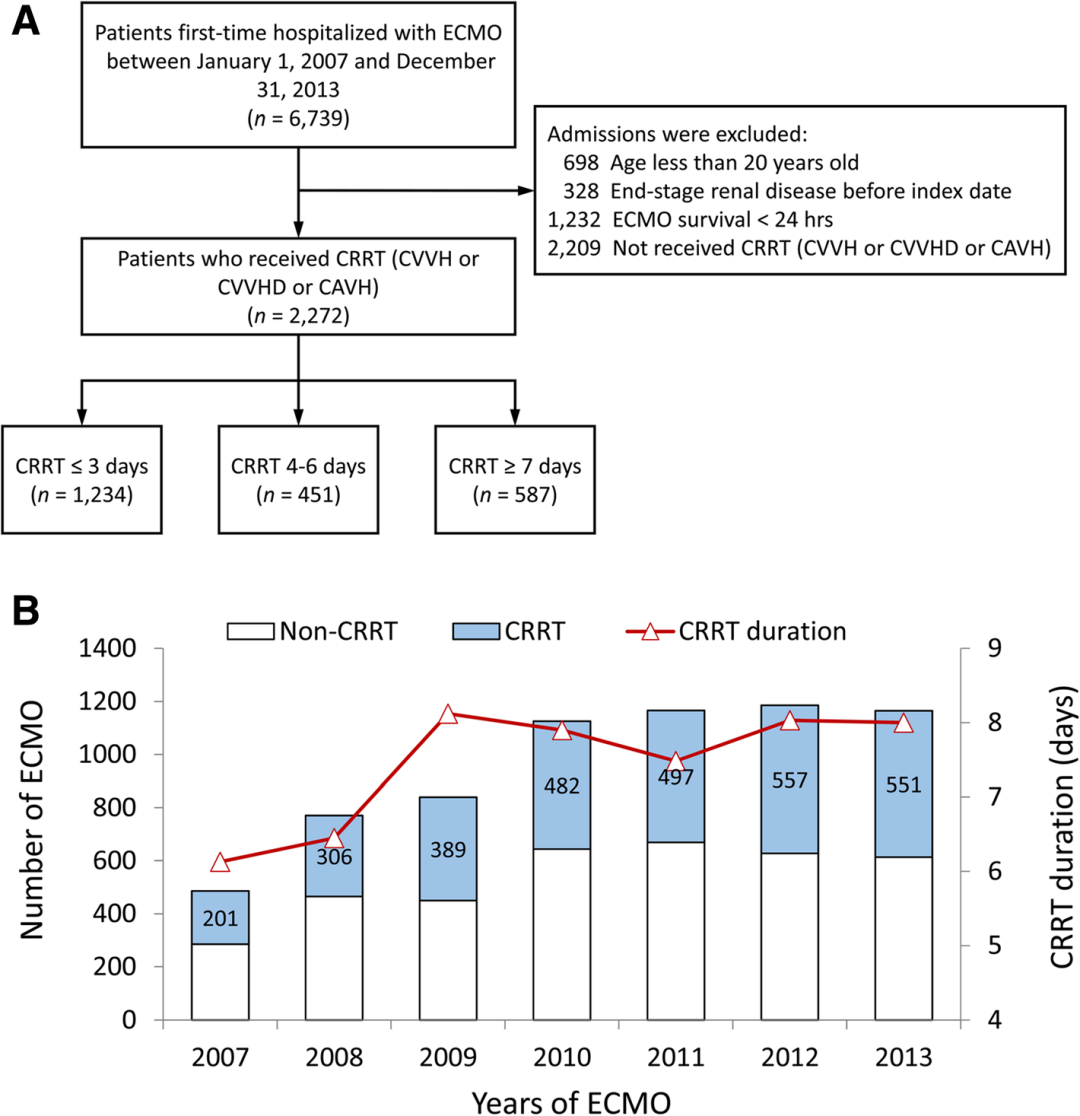
**a** Inclusion criteria. **b** Number of ECMO and CRRT admissions and CRRT duration days. CRRT, continuous renal replacement therapy; ECMO, extracorporeal membrane oxygenation

Figure 1a shows the flowchart of patient inclusion. Between January 1, 2007, and December 31, 2013, we identified 6739 patients hospitalized with first-time ECMO. After excluding patients who were younger than 20 years, had ESRD before index date, survived less than 24 h after ECMO, and did not receive CRRT, 2272 patients who received first-time ECMO and CRRT were eligible for the final analysis. These patients were further divided into three groups based on CRRT duration (≤ 3 days, 4–6 days, and ≥ 7 days). As shown in Fig. 1b, the proportion of patients who received CRRT during ECMO hospitalization and the mean duration of CRRT both gradually increased from 2007 to 2013 (*p* trend = 0.001 and 0.023, respectively).

The baseline patient characteristics are shown in Table 1. The mean age was 57.8 years with a male predominance (71.8%). The patients who underwent CRRT for ≤3 days had a higher prevalence of myocardial infarction. The distribution of ECMO indications differed among the three groups (*p* = 0.007). A cardiovascular cause of ECMO was more frequent in the CRRT ≤3 days group, whereas a respiratory cause was more common in the CRRT ≥7 days group. There were no significant differences in the patients’ age, sex, economic status, comorbidities, or follow-up duration or in the urbanization level of their residence area or the ECMO volume of their hospitals.

The overall survival to discharge was 25%, with a mean ECMO duration of 5.9 days, ICU stay of 18.1 days, and hospitalization of 26.4 days. The patients who received CRRT for ≥7 days had better in-hospital survival but longer durations on ECMO, on ventilator, in ICU, and in hospital than did those who received CRRT for ≤3 days. In-hospital mortality did not differ between the patients who received CRRT for 4–6 days and ≥ 7 days (see Additional file 1: Table S2).

**Fig. 2:**
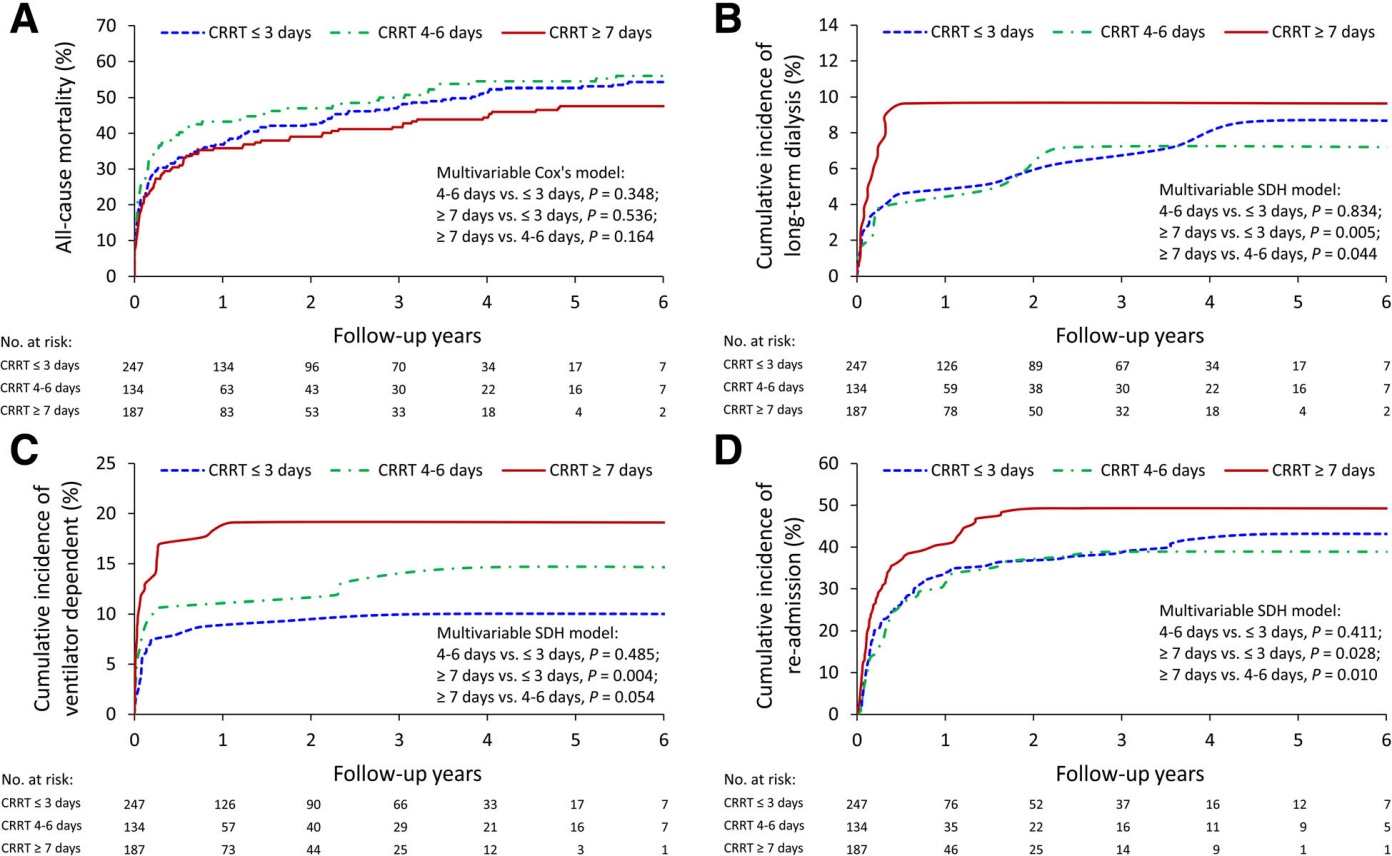
Survival rates for all-cause mortality (**a**) and cumulative incidences of ESRD (**b**), ventilator dependency (**c**), and readmission (**d**) for patients who survived the index hospitalization. CRRT, continuous renal replacement therapy; SDH, subdistribution hazard

A total of 568 patients survived the index hospitalization. The long-term outcomes were analyzed and are shown in Table 2. The mean follow-up duration was 1.62 years (SD = 1.80 years). After the adjustment of covariates, the three groups did not significantly differ in overall survival after discharge. The patients who received CRRT for ≥7 days had a higher risk of ESRD than did those who received CRRT for ≤3 days (adjusted hazard ratio [aHR] 3.46, 95% confidence interval [CI] 1.47–8.14) and 4–6 days (aHR 3.10, 95% CI 1.03–9.29). The incidence of ventilator dependence was higher in the patients who received CRRT for ≥7 days than in those who received it for ≤3 days (aHR 2.45, 95% CI 1.32–4.54). The CRRT ≥7 days group also had higher readmission rates than the other two groups did (aHR 3.46, 95% CI 1.47–8.14 vs. ≤ 3 days and aHR 3.10, 95% CI 1.03–9.29 vs. 4–6 days). The survival rates for all-cause mortality as well as the cumulative incidences of ESRD, ventilator dependency, and readmission are depicted in Fig. 2a–d. The CRRT ≥7 days group showed a trend toward more long-term dialysis when they were compared with patients who suffered AKI but did not receive CRRT (aHR 2.72 [0.94–7.87], *p* = 0.066, Additional file 1: Figure S1).

Notably, the risks of all outcomes became non-significant among all three groups when they were adjusted for the presence of sepsis, respiratory failure, and massive blood transfusion during hospitalization, the length of hospital and ICU stay, duration on ECMO and ventilator support. One explanation for this is that a collinearity may exist between the study group (CRRT group duration) and some of these in-hospital outcomes, such as ECMO duration, ventilator duration, and the length of hospital and ICU stay. For example, patients with longer CRRT durations might have longer ECMO or ventilator durations. Accordingly, the comparison of the three CRRT duration groups yielded nonsignificant results when we adjusted for the in-hospital outcomes that may be highly correlated to the study variable of primary interest.

For cross-comparing validation, we conducted a chart review in our hospital and obtained the data of 151 patients who underwent first-time ECMO during 2011–2012. In the validation of CRRT therapy, the positive predictive value was 92.1% and the negative predictive value was 90.7% (see Additional file 1: Figure S2). In the validation of ECMO indication, the Kappa agreement coefficient was 0.765 (95% CI: 0.673–0.857), indicating substantial agreement between the NHIRD and chart review (see Additional file 1: Figure S3) [13].

## Discussion

To the best of our knowledge, this is the first nationwide cohort study to investigate the relationship between CRRT duration and long-term outcomes in patients undergoing first-time ECMO. The overall hospital survival rate is 25% among patients receiving ECMO and CRRT, which is lower than the 35% survival rate on discharge among patients receiving only ECMO in a similar population [11]. This is not surprising, because AKI, particularly in patients who require dialysis therapy, is an independent risk factor of death for patients receiving ECMO [8]. Chen et al. found that the outcomes were worse in ECMO patients who had dialysis requiring AKI than in those with AKI not requiring dialysis [14]. Furthermore, Antonucci et al. reported that the use of CRRT was not associated with increased mortality in patients who received ECMO support [15].

The most critical finding in our study is that the ECMO patients who received CRRT for ≤3 days had the highest in-hospital mortality, but that the survivors had lower rates of ESRD and ventilator dependency during long-term follow-up. This contradicts the findings of previous studies in which patients who received CRRT for longer durations had poorer outcomes. Wald et al. reported that survivors in a mixed ICU population had a shorter duration on CRRT (7 days) compared with non-survivors (13 days) [16]. Tatum et al. reported 100% mortality among patients in a surgical ICU who underwent CRRT for ≥7 days [10]. Neither of those two studies included patients who received ECMO therapy. By contrast, our study focused on patients with more severe conditions that require ECMO support; therefore, the characteristics of our patients differed from those of the aforementioned research. In our study, more patients who underwent CRRT for ≤3 days received ECMO for cardiovascular causes than did their counterparts who underwent CRRT for 4–6 days or ≥ 7 days. These patients may have the worst hemodynamic status among patients receiving ECMO support (e.g., myocardial infarction or myocarditis). Both ECMO and CRRT are used to support the catastrophic disease course; thus, the higher mortality rate of CRRT of ≤3 days might reflect the uncontrolled disease This is in line with a previous study reporting that one of the risk factors of early mortality in patients receiving CRRT is a higher dose of vasopressors [17]. These patients did not survive the early stage of treatment; therefore, the duration of CRRT appears shorter.

In addition, compared with patients who received CRRT for ≤3 days and 4–6 days, more patients who spent ≥7 days on CRRT received ECMO because of respiratory failure and more frequently had sepsis during hospitalization. The longer CRRT duration implicates a delayed recovery of AKI and hemodynamic status, which may lead to lifelong dialysis dependency. This is consistent with a previous study, which demonstrated that patients with no recovery from AKI had worse renal outcomes, such as the doubling of serum creatinine as well as chronic kidney disease and ESRD [18]. These patients also share some risk factors for prolonged ventilator dependence, including intubation for respiratory failure and the presence of sepsis [19–22]. We postulate that the patients who received CRRT for ≥7 days may have survived the early period via intensive support with ECMO and CRRT, but that they were eventually left with more disabilities because of the delayed recovery of general condition.

We also revealed increased ECMO use, increased CRRT use in patients receiving ECMO, and longer CRRT duration across the study period. Similar trends have been noted in previous studies in Taiwan, South Korea, and the US [23–25]. Using CRRT in patients receiving ECMO helps maintain fluid and electrolyte balance and possibly benefits survival. However, compared with patients who receive ECMO alone, those who received ECMO and CRRT have longer hospital stays and higher expenditure [26]. Hsu et al. reported higher medical expenditure and longer hospital stays among patients receiving ECMO for respiratory failure than among those with other etiologies (cardiac, trauma, or other causes) in Taiwan [23]. This implies that patients receiving ECMO because of respiratory causes may take longer to recover. Similarly, our study found that medical expenditure was higher for the patients who received CRRT for ≥7 days, which can be explained by the longer duration of ECMO, CRRT, and hospital and ICU stay.

The current study has several limitations. First, the claim database could not provide the ICU severity score, therefore, we are unable to adjust these scores directly. Although these scoring systems are extensively used in patients receiving ECMO support, there are studies reported that the presence of AKI may have similar predictability with the Sequential Organ Failure Assessment Score or the Acute Physiology and Chronic Health Evaluation II score [27, 28]. This imply that AKI is a strong component in predicting outcome in patient receiving ECMO, and in the presence of AKI, the predictability of these scores may decrease. Comparing with another frequently used scoring system, Simplified Acute Physiology Score, our study have already adjusted for multiple factors including patients’ age, comorbidities, duration of hospital or ICU stay, cardiac or trauma surgery, and the presence of sepsis, all of them are important component of the score. The adjustment may partially compensate the absence of severity score in this database. Second, the indication of ECMO initiation was analyzed indirectly by using the NHI reimbursement code for the procedure and ICD-9 diagnostic codes. This may lead to some misclassification among different groups. To confirm the reliability of this information, we performed a cross-comparison validation; the results, as mentioned, indicated substantial agreement. Third, the ECMO circuit (i.e., veno-venous or veno-arterial) could not be distinguished from the NHIRD data. This limitation also applied to the indication of CRRT initiation. Thus, we were unable to analyze how different ECMO circuits and CRRT indications may have affected long-term patient survival. Finally, pediatric patients were excluded from this study; hence, the experience of pediatric patients receiving ECMO and CRRT must be evaluated in future research.

## Conclusions

Our study found similar long-term survival but increased long-term ESRD and ventilator dependency among ECMO patients who underwent CRRT for ≥7 days compared with counterparts who received the treatment for shorter periods. These results support concerns that the aggressive form of life support may maintain patient survival but do so at the cost of long-term disabilities and, consequently, a lower quality of life along with increased financial burdens on health care systems as well as patients and their families. For ECMO patients receiving CRRT for ≥7 days, clinicians may need to discuss the related long-term morbidities with patients and their representatives.

## Additional file

Additional file 1: Description of NHIRD. **Table S1.** ICD-9-CM code used for diagnosis in the current study. **Table S2.** In hospital outcomes. **Figure S1.** Comparison of long-term dialysis among AKI patients who did not receive CRRT and those received CRRT for ≤3 days, 4–6 days and ≥ 7 days. **Figure S2**. Validation of CRRT. **Figure S3**. Validation of ECMO indication (DOCX 204 kb)

## Data Availability

Data Availability Statement: The data underlying this study is from the National Health Insurance Research Database (NHIRD), which has been transferred to the Health and Welfare Data Science Center (HWDC). Interested researchers can obtain the data through formal application to the HWDC, Department of Statistics, Ministry of Health and Welfare, Taiwan (http://dep.mohw.gov.tw/DOS/np-2497-113.html).

## Abbreviations

AKI: Acute kidney injury
CAVH: Continuous arterio-venous hemofiltration
CI: Confidence interval
CRRT: Continuous renal replacement therapy
CVVH: Continuous veno-venous hemofiltration
CVVHD: Continuous veno-venous hemodialysis
ECMO: Extracorporeal membrane oxygenation
ESRD: End-stage renal disease
ICD-9-CM: International Classification of Diseases, Ninth Revision, Clinical Modification
NHIRD: National Health Insurance Research Database
SD: Standard deviation
