# Supplementary material for: Analysis of survival after initiation of continuous renal replacement therapy in patients with extracorporeal membrane oxygenation
Source: BMC Nephrol. 2019 Aug 14;20:318. doi: 10.1186/s12882-019-1516-6 (PMC6694695; doi:10.1186/s12882-019-1516-6)
Supplement: Supplementary file 1 — Description of NHIRD. Table S1. ICD-9-CM code used for diagnosis in the current study. Table S2. In hospital outcomes. Figure S1. Comparison of long-term dialysis among AKI patients who did not receive CRRT and those received CRRT for ≤3 days, 4–6 days and ≥ 7 days. Figure S2. Validation of CRRT. Figure S3. Validation of ECMO indication (DOCX 204 kb) [file 12882_2019_1516_MOESM1_ESM.docx]

**Description about the NHIRD**

The database is derived from the NHI program, which has provided compulsory universal health insurance in Taiwan since 1995, with a coverage rate of approximately 99.6% coverage. The NHIRD contains comprehensive healthcare records and offers encrypted datasets for medical research. The NHI started reimbursing ECMO therapy in December 2002 and CRRT for post-cardiac surgery AKI in April 2006. The indications of all medications, procedures, and surgeries are subjected to medical claims review and auditing by the NHI administrators. Hospitals must provide relevant medical records to support their use of medications and surgeries. Any treatment with insufficient indications will not be reimbursed. Certain severe diseases, including ESRD and ventilator dependency, were identified in this study according to Catastrophic Illness Certificates (CICs). Patients with ESRD must receive lifelong dialysis therapy with a permanent vascular access to qualify the CIC. Patients applying for a CIC for ventilator dependency must receive at least 6 hours of positive ventilator a day for at least 18 consecutive days. CIC holders are exempt from paying insurance premiums and copayments. All CIC applications are carefully reviewed by the Taiwan Bureau of National Health Insurance; thus, CIC data are accurate and reliable.

Reference

1. Chen SW, Tsai FC, Lin YS, Chang CH, Chen DY, Chou AH, Chen TH: **Long-term outcomes of extracorporeal membrane oxygenation support for postcardiotomy shock**. *J Thorac Cardiovasc Surg* 2017, **154**(2):469-477 e462
2. Chen SW, Chang CH, Lin YS, Wu VC, Chen DY, Tsai FC, Hung MJ, Chu PH, Lin PJ, Chen TH: **Effect of dialysis dependence and duration on post-coronary artery bypass grafting outcomes in patients with chronic kidney disease: A nationwide cohort study in Asia**. *International journal of cardiology* 2016, **223**:65-71

**Supplementary Table 1**. ICD-9-CM code used for diagnosis in the current study

| Variable | ICD-9-CM code |
| --- | --- |
| End stage renal disease | 585.xx (Catastrophic illness card) |
| Chronic kidney disease | 580.xx–589.xx, 403.xx–404.xx, 016.0x, 095.4x, 236.9x, 250.4x, 274.1x, 442.1x, 447.3x, 440.1x, 572.4x, 642.1x, 646.2x, 753.1x, 283.11, 403.01, 404.02, 446.21 |
| Diabetes mellitus | 250.xx |
| Hypertension | 401.xx–405.xx |
| Heart failure | 428.xx |
| Coronary artery disease | 410.xx–414.xx |
| Prior myocardial infarction | 410.xx, 412.xx |
| Atrial fibrillation | 427.31 |
| Peripheral arterial disease | 440.0x, 440.2x, 440.3x, 440.8x, 440.9x, 443.xx, 444.0x, 444.22, 444.8x, 447.8x, 447.9x |
| Stroke | 430.xx–437.xx |
| Chronic obstructive pulmonary disease | 491.xx, 492.xx, 496.xx |
| Liver cirrhosis | 571.2x, 571.5x, 571.6x |
| Malignancy | 140.xx–208.xx (Catastrophic illness card) |
| Ventilator dependent | 518.xx (Catastrophic illness card) |
| ECMO indication |  |
| Cardiogenic shock | 411.0, 411.1, 411.8, 411.81, 411.89, 412, 413.0, 413.1, 413.9, 414.0, 414.00, 414.01, 414.06, 414.2, 414.3, 414.4, 414.8, 414.9, V458.1, V458.2, 415.0, 415.1, 415.12, 415.13, 415.19, 416.0, 416.1, 416.2, 416.8, 416.9, 417.0, 417.1, 417.8, 417.9, V125.5, 427.0, 427.1, 427.2, 427.31, 427.32, 427.60, 427.61, 427.69, 427.81, 427.89, 427.9, 785.0, 785.1, 427.41, 427.42, 427.5, 398.91, 428.0, 428.1, 428.20, 428.21, 428.22, 428.23, 428.30, 428.31, 428.32, 428.33, 428.40, 428.41, 428.42, 428.43 and 428.9 |
| Myocarditis | 328.2, 364.0, 364.1, 364.2, 364.3, 742.0, 742.1, 742.2, 742.3, 112.81, 115.03, 115.04, 115.13, 115.14, 115.93, 115.94, 130.3, 391.0, 391.1, 391.2, 391.8, 391.9, 392.0, 393, 398.0, 398.90, 398.99, 420.0, 420.90, 420.91, 420.99, 421.0, 421.1, 421.9, 422.0, 422.90, 422.91, 422.92, 422.93, 422.99, 423.0, 423.1, 423.2, 423.3, 423.8, 423.9, 425.0, 425.1, 425.11, 425.18, 425.2, 425.3, 425.4, 425.7, 425.8, 425.9 and 429.0 |
| Acute myocardial infarction | 410.0, 410.00, 410.01, 410.02, 410.1, 410.10, 410.11, 410.12, 410.2, 410.20, 410.21, 410.22, 410.3, 410.30, 410.31, 410.32, 410.4, 410.40, 410.41, 410.42, 410.5, 410.50, 410.51, 410.52, 410.6, 410.60, 410.61, 410.62, 410.7, 410.70, 410.71, 410.72, 410.8, 410.80, 410.81, 410.82, 410.9, 410.90, 410.91 and 410.92 |
| Respiratory | 020.0, 003.22, 020.3, 020.4, 020.5, 020.8, 020.9, 021.2, 021.8, 021.9, 022.1, 022.8, 022.9, 023.0, 023.1, 023.2, 023.3, 023.8, 023.9, 024, 025, 026.0, 026.9, 027.0, 027.1, 027.2, 027.8, 027.9, 030.0, 030.1, 030.2, 030.3, 030.8, 030.9, 031.0, 031.2, 031.8, 031.9, 032.0, 032.1, 032.2, 032.3, 032.89, 032.9, 033.0, 033.1, 033.8, 033.9, 034.0, 034.1, 036.3, 036.81, 036.89, 036.9, 037, 039.1, 039.2, 039.3, 039.4, 039.8, 039.9, 040.0, 040.1, 040.2, 040.3, 040.42, 040.81, 040.82, 040.89, 041.0, 041.0, 041.1, 041.2, 041.3, 041.4, 041.5, 041.9, 041.1, 041.10, 041.11, 041.12, 041.19, 041.2, 041.3, 041.4, 041.41, 041.42, 041.43, 041.49, 041.5, 041.6, 041.7, 041.8, 041.81, 041.82, 041.83, 041.84, 041.85, 041.86, 041.89, 041.9, 052.1, 055.1, 073.0, 083.0, 112.4, 114.0, 114.4, 114.5, 115.5, 115.15, 115.95, 130.4, 136.3, 390, 392.9, 460, 461.0, 461.1, 461.2, 461.3, 461.8, 461.9, 462, 464.0, 464.0, 464.1, 464.10, 464.11, 464.20, 464.21, 464.30, 464.31, 464.4, 464.50, 464.51, 465.0, 465.8, 465.9, 473.0, 473.1, 473.2, 473.3, 473.8, 473.9, 480.0, 480.1, 480.2, 480.3, 480.8, 480.9, 481, 482.0, 482.1, 482.2, 482.3, 482.30, 482.31, 482.32, 482.39, 482.4, 482.40, 482.41, 482.42, 482.49, 482.8, 482.81, 482.82, 482.83, 482.84, 482.89, 482.9, 483, 483.0, 483.1, 483.8, 484.1, 484.3, 484.5, 484.6, 484.7, 484.8, 485, 486, 487.0, 487.1, 487.8, 488, 488.0, 488.1, 488.2, 488.9, 488.1, 488.11, 488.12, 488.19, 488.81, 488.82, 488.89, 495.0, 495.1, 495.2, 495.3, 495.4, 495.5, 495.6, 495.7, 495.8, 495.9, 500, 501, 502, 503, 504, 505, 506.0, 506.1, 506.2, 506.3, 506.4, 506.9, 507.1, 507.8, 508.0, 508.1, 508.2, 508.8, 508.9, 510.0, 510.9, 511.0, 511.1, 511.8, 511.89, 511.9, 512.0, 512.8, 512.81, 512.82, 512.83, 512.84, 512.89, 513.0, 517.1, 517.3, 518.0, 518.1, 518.2, 518.5, 518.51, 518.52, 518.53, 518.81, 518.82, 518.83, 518.84, 784.91, 795.3, 795.31, 795.39, 799.1, V090, V091, V092, V093, V094, V095.0, V095.1, V096, V097.0, V097.1, V098.0, V098.1, V099.0, V099.1, V120.4, V461, V461.1, V461.2, V461.3, V461.4 and V462 |
| Trauma | 800.xx–994.xx |

ICD-9-CM, International Classification of Diseases, Ninth Revision, Clinical Modification; ECMO, extracorporeal membrane oxygenation.

**Supplementary Table 2**. In hospital outcomes

|  | Total  (*n* = 2,272) | CRRT ≤ 3 days  (*n* = 1,234) | CRRT 4-6 days  (*n* = 451) | CRRT ≥ 7 days  (*n* = 587) | *P* |
| --- | --- | --- | --- | --- | --- |
| Outcome |  |  |  |  |  |
| Categorical parameter |  |  |  |  |  |
| In-hospital mortality | 1,704 (75.0) | 987 (80.0) | 317 (70.3)a | 400 (68.1)a | <0.001 |
| New onset any stroke | 116 (5.1) | 54 (4.4) | 28 (6.2) | 34 (5.8) | 0.217 |
| New onset ischemic stroke | 75 (3.3) | 36 (2.9) | 17 (3.8) | 22 (3.7) | 0.536 |
| New onset hemorrhagic stroke | 43 (1.9) | 19 (1.5) | 11 (2.4) | 13 (2.2) | 0.390 |
| Sepsis | 625 (27.5) | 296 (24.0) | 117 (25.9) | 212 (36.1)ab | <0.001 |
| Fasciotomy or amputation | 54 (2.4) | 26 (2.1) | 11 (2.4) | 17 (2.9) | 0.584 |
| Respiratory failure | 417 (18.4) | 131 (10.6) | 78 (17.3)a | 208 (35.4)ab | <0.001 |
| IABP | 1,012 (44.5) | 546 (44.2) | 221 (49.0) | 245 (41.7) | 0.063 |
| Massive blood transfusion, PRBC > 10 Units | 1,564 (68.8) | 750 (60.8) | 323 (71.6)a | 491 (83.6)ab | <0.001 |
| Continuous parameter |  |  |  |  |  |
| PRBC amount | 21.8±18.7 | 18.7±16.5 | 21.3±17.5a | 28.8±21.7ab | <0.001 |
| FFP amount | 17.6±24.4 | 14.8±21.7 | 16.9±21.4 | 24.0±30.2ab | <0.001 |
| Platelet amount | 14.8±25.0 | 11.3±21.3 | 14.6±23.3a | 22.2±31.0ab | <0.001 |
| ECMO support duration (days) | 5.9±3.8 | 4.8±3.3 | 6.1±2.8a | 7.9±4.4ab | <0.001 |
| Ventilator (days) | 16.9±18.9 | 12.1±15.1 | 16.7±18.2a | 27.2±22.2ab | <0.001 |
| ICU duration (days) | 18.1±19.5 | 13.1±16.1 | 17.7±18.7a | 29.1±22.0ab | <0.001 |
| Hospital stays (days) | 26.4±31.3 | 19.7±27.0 | 25.0±28.4a | 41.4±36.5ab | <0.001 |
| Inpatient medical expenditure (NTD×10^4^) | 105.0±80.5 | 83.6±66.6 | 101.2±69.1a | 152.7±94.1ab | <0.001 |

IABP, intra-aortic balloon pump; PRBC, packed red blood cells; FFP, fresh frozen plasma; ICU, intensive care unit; NTD, New Taiwan Dollar;

a indicates *P* < 0.05 versus CRRT ≤ 3 days and b indicates *P* < 0.05 versus CRRT 4-6 days in the Bonferroni multiple comparisons.

**Supplementary Figure 1**. Comparison of long-term dialysis among AKI patients who did not receive CRRT and those received CRRT for ≤ 3 days, 4-6 days and ≥ 7 days.

**
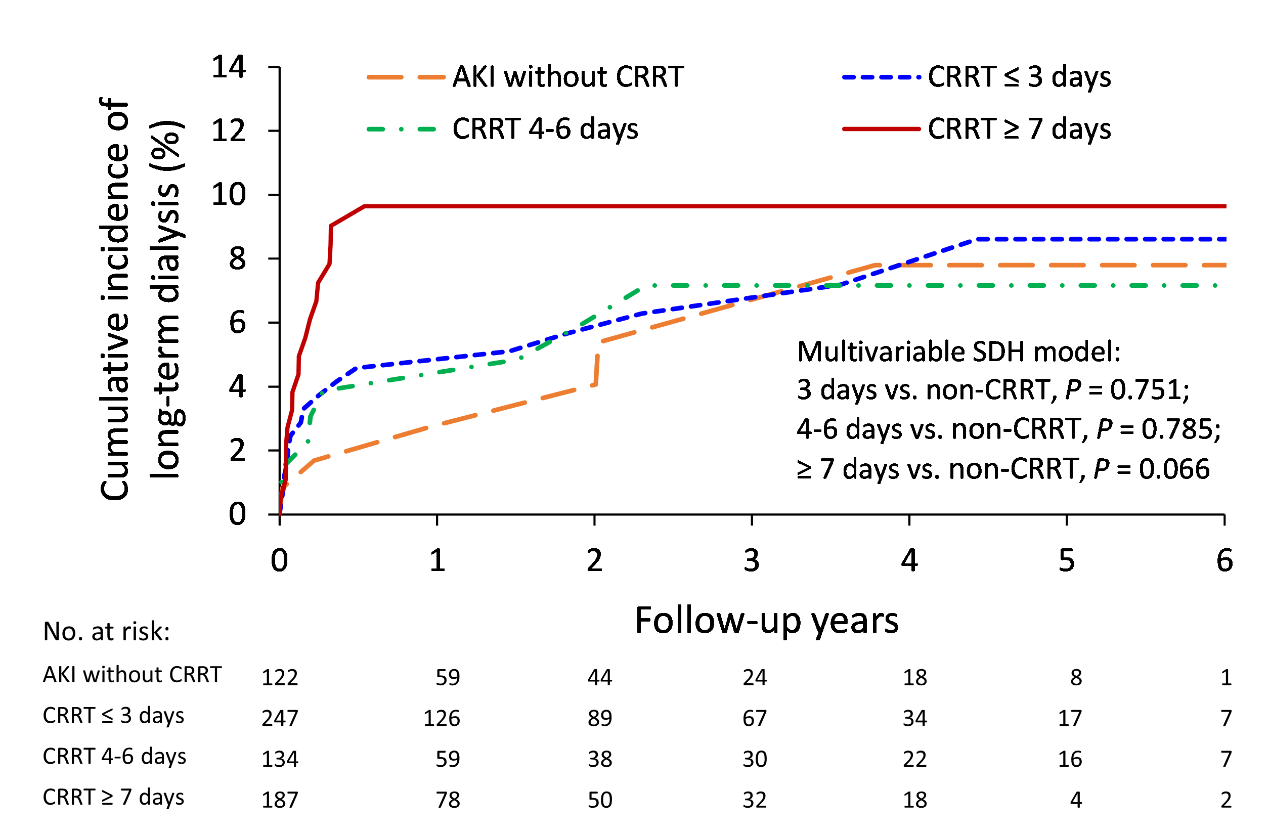
**

**Supplementary Figure 2**. Validation of CRRT

|  |  | Linkou CGMH | |  |
| --- | --- | --- | --- | --- |
|  |  | + | - |  |
| NHIRD | + | 70 | 6 | 76 |
|  | - | 7 | 68 | 75 |
|  |  | 77 | 74 |  |

Positive predicted value = 92.11%, negative predicted value = 90.67%;

NHIRD, national health insurance research database; CGMH, Chang Gung Memorial Hospital.

**Supplementary Figure 3**. Validation of ECMO indication

|  |  | Linkou CGMH | | | | |  |
| --- | --- | --- | --- | --- | --- | --- | --- |
|  |  | Cardiovascular | Post-cardiotomy shock | Respiratory | Trauma | Others |  |
| NHIRD | Cardiovascular | 19 | 2 | 2 | 0 | 2 | 25 |
|  | Post-cardiotomy shock | 5 | 66 | 2 | 3 | 5 | 81 |
|  | Respiratory | 1 | 1 | 28 | 1 | 3 | 34 |
|  | Trauma | 2 | 0 | 1 | 3 | 1 | 7 |
|  | Others | 1 | 0 | 2 | 0 | 1 | 4 |
|  |  | 28 | 69 | 35 | 7 | 12 |  |

Kappa agreement coefficient = 0.765, 95% confidence interval = 0.673 to 0.857;

NHIRD, National Health Insurance Research Database; CGMH, Chang Gung Memorial Hospital.
